# Comparative efficacy of neuromodulation and structured exercise program on pain and muscle oxygenation in fibromyalgia patients: a randomized crossover study

**DOI:** 10.3389/fphys.2024.1414100

**Published:** 2024-07-23

**Authors:** Alejandro Rubio-Zarapuz, María Dolores Apolo-Arenas, José Francisco Tornero-Aguilera, Jose A. Parraca, Vicente Javier Clemente-Suárez

**Affiliations:** ^1^ Faculty of Sports Sciences, Universidad Europea de Madrid, Madrid, Spain; ^2^ Department of Medical Surgical-Therapy, Faculty of Medicine and Health Sciences, Universidad de Extremadura, Badajoz, Spain; ^3^ Research Group PhysioH, University of Extremadura, Badajoz, Spain; ^4^ Departamento de Desporto e Saúde, Escola de Saúde e Desenvolvimento Humano, Universidade de Évora, Évora, Portugal; ^5^ Comprehensive Health Research Centre (CHRC), University of Évora, Évora, Portugal; ^6^ Grupo de Investigación en Cultura, Educación y Sociedad, Universidad de la Costa, Barranquilla, Colombia

**Keywords:** fibromyalgia, neuromodulation therapy, exercise program, muscle oxygenation, pain modulation, HIIT, EXOPULSE Mollii suit

## Abstract

**Introduction:** This study investigates the comparative efficacy of neuromodulation therapy using the EXOPULSE Mollii Suit and a structured exercise program in pain modulation and muscle oxygenation in Fibromyalgia patients.

**Methods:** A randomized, crossover, longitudinal, and experimental study design was employed, involving 10 female Fibromyalgia patients. Participants were subjected to two distinct treatment modalities: neuromodulation therapy with the EXOPULSE Mollii Suit and a strength-based High-Intensity Interval Training (HIIT) exercise program, each conducted over 16 sessions. Outcome measures included pain severity, assessed using the Numeric Rating Scale (NRS), and muscle oxygenation variables measured via Near-Infrared Spectroscopy (NIRS).

**Results:** Both interventions demonstrated significant reductions in NRS scores and improvements in muscle oxygenation. However, the exercise program yielded more pronounced long term basal adaptations in muscle oxygenation compared to the neuromodulation therapy.

**Discussion:** The findings underscore the potential of integrating non-pharmacological treatments, particularly structured exercise programs, in managing Fibromyalgia. While neuromodulation therapy presents a viable alternative, the exercise regimen’s capacity to induce basal muscle oxygenation adaptations suggests its superiority in addressing the complex symptoms of Fibromyalgia. Furthermore, these therapeutic approaches may enhance patients’ vocational values and employability opportunities by improving their functional capabilities and overall quality of life.

## 1 Introduction

Fibromyalgia is recognized as a chronic disorder characterized by widespread musculoskeletal pain, accompanied by fatigue, sleep and autonomic disturbances, cognitive impairment, heightened sensitivity to external stimuli, various somatic symptoms, and psychiatric comorbidities (Antunes and Marques, 2022; Gyorfi et al., 2022). It is identified as one of the three most prevalent musculoskeletal conditions globally, following lumbar pain and osteoarthritis, with a global prevalence estimated at 2%–3% (Gyorfi et al., 2022). This prevalence escalates to approximately 4.7% within Western European demographics (Gilheaney and Chadwick, 2024). The condition exhibits a predilection towards the female population, presenting a female-to-male ratio of 3:1 (Kocyigit and Akyol, 2022), and demonstrates an increased prevalence with advancing age, notably peaking between the ages of 50 and 60 (Wilgen et al., 2024).

The nature, location, and intensity of the musculoskeletal pain experienced by individuals with Fibromyalgia vary significantly, influenced by occupational roles, comorbid conditions, environmental factors such as temperature and pressure, alongside physical or mental stressors (Pinto et al., 2023). These patients may also suffer from both physical and mental fatigue, which can range from mild lethargy to states of exhaustive fever-like conditions (Zambolin et al., 2022). Commonly, individuals with Fibromyalgia encounter insomnia (Catalá et al., 2023), cognitive dysfunction including memory deficits (Millar et al., 2024), as well as depression and anxiety (Yepez et al., 2022), headaches (Dong et al., 2023), gastrointestinal disturbances (Gilheaney and Chadwick, 2024), genitourinary disorders (Ayan et al., 2023), morning stiffness (Plaut, 2022), and autonomic dysregulation, as evidenced by xerostomia and xerophthalmia, blurred vision, or photophobia (Gyorfi et al., 2022). Additionally, this patient population often experiences negative emotional states and a pervasive sense of distress (Romeo et al., 2022), potentially contributing to the higher prevalence of psychiatric disorders, with anxiety disorders reported in 60% of patients and depression ranging between 14% and 36%, in contrast to a 6.6% prevalence within the general population (Sadr et al., 2023). Moreover, recent findings have elucidated mitochondrial dysfunction in Fibromyalgia patients (Gerdle et al., 2020), demonstrating significantly reduced muscle oxygen saturation levels as low as 20%, compared to a normal range of approximately 75% (Villafaina et al., 2023). The etiology of this reduction, whether due to a deficiency in mitochondrial energy production or an increased energy demand by muscle fibers, remains to be fully determined (Rubio-Zarapuz et al., 2023; Rubio-Zarapuz et al., 2024).

The diagnosis of Fibromyalgia presents considerable challenges due to the absence of overt clinical signs, distinct from other rheumatic diseases, and the lack of definitive biomarkers for assessment (Dizner-Golab et al., 2023). Over the last three decades, five distinct sets of classification and diagnostic criteria have been developed (Alsiri et al., 2023). Although a clear pathogenesis for Fibromyalgia has not been fully established, potential etiological factors include genetic predispositions, profound psychological trauma, peripheral inflammation, and dysregulation of central pain processing mechanisms, resulting in what is known as nociplastic pain (Bidari and Ghavidel-Parsa, 2022). Recent studies involving microRNA, proteome, and metabolome analyses have yielded promising results in disease detection (Alsiri et al., 2023).

The complexity of Fibromyalgia necessitates a comprehensive, multidisciplinary approach to management, integrating pharmacological and psychological therapies, patient education, exercise, and dietary modifications (Rhodes et al., 2023). Pharmacological interventions focus on analgesia 34, with certain centrally acting drugs, such as antidepressants and anticonvulsants, showing efficacy by modulating pain-inhibitory neurotransmitters, dorsal horn sensitization, and systemic hyperexcitability (Migliorini et al., 2022). However, only a quarter of patients achieve a 30% reduction in pain symptoms through antidepressant therapy (Aster et al., 2022). Given the heterogeneity of the disease’s manifestation, pharmacological treatment is not standardized, leading to the utilization of various other medications including muscle relaxants (Martinez and Guimarães, 2024), analgesics (Valladales-Restrepo et al., 2023), hypnotic and antipsychotic drugs (Ozgunay et al., 2024), and cannabinoids (Strand et al., 2023). Despite extensive research, no single pharmacological agent consistently benefits more than half of the patient population treated (Hong-Baik et al., 2023). Cognitive-behavioral therapy, aimed at developing effective coping strategies, has shown superior outcomes in improving pain, physical function, and mood among Fibromyalgia patients compared to other interventions (Lee et al., 2023). Education plays a pivotal role in enabling patients to understand the chronic nature of their condition and to take an active part in its management (Duhn et al., 2023).

Additionally, while pharmacological treatments exert a more targeted effect on patient physiology, non-pharmacological interventions offer a broad, multifaceted impact that is difficult to achieve through pharmacological means alone (Hong-Baik et al., 2023). A variety of non-pharmacological treatments, including spa therapy (Fioravanti et al., 2022), Tai Chi, Qigong, yoga (Neelapala et al., 2023), mindfulness practices (Gordon et al., 2023), hypnosis (Ozgunay et al., 2024), acupuncture (Han et al., 2023), thermal or cryotherapy (Legrand et al., 2023), hyperbaric oxygen therapy (Chen et al., 2023), and transcranial electrical and magnetic stimulation (Toh et al., 2022), have been explored. Neuromodulation, either through localized transcutaneous electrical nerve stimulation (TENS) systems (Jamison et al., 2021; Cheng et al., 2023) or the use of the EXOPULSE Mollii^®^ suit (Riachi et al., 2023; Rubio-Zarapuz et al., 2023; Rubio-Zarapuz et al., 2024), has shown to offer beneficial effects on pain perception, muscle oxygenation, parasympathetic activity, and overall functionality in Fibromyalgia patients. Exercise and nutritional strategies are recommended as initial interventions over pharmacological treatments, focusing on aerobic and strength training, weight management, and dietary adjustments (Carrasco-Querol et al., 2023). These interventions are associated with improvements in posture and wellbeing, reduction in obesity-induced inflammation, and enhancement of pain management and functional outcomes (Hong-Baik et al., 2023). On this line, a 60-min strength training session has shown promising effects in breathing parameter, pain perception, cortical arousal, muscle oxygenation, autonomic modulation, and overall function.

This study aims to investigate the effects of muscle oxygenation and pain perception in Fibromyalgia patients undergoing 16 sessions of treatment with the EXOPULSE Mollii suit compared to a conventional 16-session exercise training program. The hypothesize that significant differences in pain perception and muscle oxygenation levels will be discerned between baseline and post-intervention assessments across the differing treatment modalities. This hypothesis-driven approach aims to provide a more focused framework for understanding the comparative efficacy of neuromodulation therapy using the EXOPULSE Mollii Suit and a structured exercise program in Fibromyalgia patients.

## 2 Materials and methods

### 2.1 Study design

The current investigation was conducted within the Faculty of Medicine at the University of Badajoz, Spain. This research, adopting a randomized, crossover, longitudinal, and experimental framework, was elaborately constructed to assess and juxtapose the prolonged impacts of two divergent therapeutic strategies on subjects diagnosed with fibromyalgia, in strict compliance with the 2016 American College of Rheumatology (ACR) Criteria for the diagnosis of fibromyalgia (Schweiger et al., 2024).

The therapeutic interventions subjected to comparison comprised a regimen involving the application of the EXOPULSE Mollii suit (Exoneural Network, Sweden), alongside a physical exercise protocol. The process of participant recruitment spanned from September 2022 to December 2022, facilitating an exhaustive and thorough inclusion phase. Subsequently, the commencement of the intervention phase was initiated in January 2023. This designated period permitted a meticulous screening and selection procedure, guaranteeing the adherence of all enrollees to the rigorous diagnostic benchmarks for fibromyalgia as delineated by the ACR. The architecture of this study’s methodological approach was intentionally crafted to rigorously evaluate and document the immediate, short-term, and extensive long-term outcomes of the aforementioned therapeutic interventions.

### 2.2 Participants

Upon the completion of a thorough patient recruitment and evaluation phase, the present investigation successfully registered a cohort of 10 female patients afflicted with fibromyalgia (mean age: 51.6 ± 7.18 years; mean weight: 68.5 ± 8.26 kg; mean height: 160 ± 3.80 cm; Body Mass Index (BMI): 26.7 ± 2.79 kg/m^2^). Eligibility for inclusion mandated adherence to several rigorous prerequisites: participants must have been formally diagnosed with fibromyalgia by a qualified rheumatologist pursuant to the criteria set forth by the ACR, with the diagnosis having been established a minimum of 3 months prior to commencement. Furthermore, inclusion was confined to female individuals aged between 18 and 67 years who possessed the ability to walk independently, devoid of reliance on assistive devices. Exclusion criteria were meticulously outlined: potential candidates were deemed ineligible if they were concurrently engaged in other clinical investigations, had undergone neuromodulation therapy, or had participated in organized exercise regimes in the 6 months leading up to the study.

Additionally, exclusion was necessitated for those who did not provide written consent, had concurrent neurological disorders or conditions that significantly impacted pain perception, had recently undergone surgical procedures or sustained musculoskeletal injuries within the preceding 6 months, presented with severe cardiovascular or respiratory conditions that contraindicated exercise, or were undergoing opioid therapy or had experienced alterations in their pain management medication regimen in the past month.

The finalized participant pool represented the maximal attainable cohort size within the limitations posed by the available scheduling opportunities, the capacities of the facilities, and the resources of the personnel involved. This meticulous selection mechanism ensured the assembled participant group stringently complied with both the operational and clinical criteria requisite for involvement, thereby enabling a controlled and methodologically sound exploration of the intervention’s impact on fibromyalgia symptoms.

### 2.3 Intervention

To fulfill the aims of this research, participants were randomly distributed into two distinct cohorts: the Suit + Exercise group and the Exercise + Suit group. The experimental framework was intricately designed to ensure procedural consistency across both cohorts. Participants underwent two sessions weekly of the designated intervention, spanning an 8-week duration. This stringent regimen culminated in a total of 16 sessions for each segment of the study. Subsequent to these sessions, a 2-week washout period was instituted to neutralize any residual effects of the treatments, a pivotal step designed to restore the participants’ physiological baseline and safeguard the integrity of outcomes in the ensuing phase. Following this interval, a second sequence of 16 sessions was administered, during which individuals were subjected to the alternative intervention, thus fulfilling the crossover methodology of the investigation. In the initial phase, individuals in the Suit + Exercise group were administered neuromodulation treatment before proceeding to the exercise protocol. In contrast, the Exercise + Suit group commenced with the exercise intervention, subsequently receiving the neuromodulation therapy. The specific interventions applied to each group are elaborated upon as follows: Table 1.

• Suit: Participants engaged in a 60-min session utilizing the EXOPULSE Mollii suit, activating all 58 electrodes at an intensity setting of 2 milliamperes (mA) and a pulse width of 30 milliseconds (ms). This protocol mirrors the treatment procedures documented in preceding research (Pennati et al., 2021; Raffalt et al., 2022; Rubio-Zarapuz et al., 2023; 2024). Each participant was assisted by a certified professional to ensure accurate placement of all electrodes. Upon correct application of the suit and attachment of the control unit, the participant was positioned supine on a massage table, at which point the suit was activated to commence the session.
• Exercise: Participants partook in a 1-h training session per meeting, initiating with a mobility warm-up, followed by a main training session integrating strength exercises and High-Intensity Interval Training (HIIT). This regimen was systematically advanced throughout the intervention period, as outlined in. The initial two sessions were dedicated to a familiarization phase, crucial for establishing a foundation for the subsequent intensive training. This phase aimed at acclimating participants to the exercise regimen, ensuring their comfort with the routines, and minimizing the risk of injury.

**TABLE 1:** Exercise intervention.

Week	Week 1		Week 2–4						Week 5–8							
Contents	Adapted Push Ups		Walking + (Isometric Dead Bug + Load on the Arms by a Band) + Adapted Push Ups						(Squat + Weight Ball Throw) + Bench Press + Dead Lift							
	Sit to Stand		Up and Down Stairs + (Isometric Dead Bug + Load on the Legs by a Band) + Glute Bridge						(Squat + Front Weight Ball Throw) + Military Press + Isometric Squat							
	1 Hand Row with Band		Walking + Both Hands Isometric Row with Band + Sit to Stand Aided by TRX						(Squat + Sideways Weight Ball Throw) + Lunges with Load + TRX Row							
	Glute Bridge		Climb Up and Down Stairs + Isometric Squat Aided by TRX + Sit to Stand						Weight Ball Slam + Loaded Step Up + Biceps Curl TRX							
Session	1	2	3	4	5	6	7	8	9	10	11	12	13	14	15	16
Sets	3	4	3		4				3				4			
Work	10		15″ + 15″ + 6	25″ + 15″ + 10		25″ + 20″ + 10		25″ + 25″ + 10	3 × 10	3 × 12	3 × 15	3 × 15	3 × 10	3 × 12	3 × 15	3 × 15
Rest	1 min								2 min							

This table outlines the progression and components of the training regimen implemented across the 16-session exercise program. It details the specific exercises, intensity levels, duration, and other relevant parameters structured to optimize participant outcomes.

### 2.4 Measurements

Outcome measures were meticulously evaluated both prior to and following the interventions at three critical junctures: the 1st, 8th, and 16th treatment sessions for each treatment modality. This multistage evaluation strategy aligns with methodologies that have been previously validated in longitudinal research, providing a robust framework for the quantification of intervention effects on study participants. The selection of variables for analysis and the assessment methodologies were informed by established protocols articulated in preceding scholarly investigations (Rubio-Zarapuz et al., 2023; Rubio-Zarapuz et al., 2024). This methodical approach not only facilitates the comparison of outcomes across different studies but also significantly enriches the body of scientific knowledge concerning the efficacy of various therapeutic approaches for managing fibromyalgia. Additionally, the adoption of standardized assessment techniques bolsters the validity of this study, ensuring that its conclusions are both credible and transferable to wider clinical settings.

#### 2.4.1 Muscle oxygen variables

Muscle oxygen saturation (SmO_2_), total hemoglobin (THb), deoxygenated hemoglobin (HHb), and oxygenated hemoglobin (O_2_Hb) values were measured using a portable NIRS sensor (Moxy, Fortiori Design LLC, Hutchinson, MN, United States) indices were quantified employing a portable Near-Infrared Spectroscopy (NIRS) sensor (Moxy, Fortiori Design LLC, Hutchinson, MN, United States), interfaced with GoldenCheetah software (version 3.4, U.S.). This instrument, validated for its reliability in both low and moderate exercise intensities for the assessment of muscle oxygen consumption [SmO_2_; Intraclass Correlation Coefficient (ICC): *r* = 0.773–0.992] (Yogev et al., 2023), was strategically positioned on the vastus lateralis muscle, midway between the greater trochanter and the lateral femoral epicondyle (Villafaina et al., 2023). To mitigate signal noise, a soft spline filter was employed using MATLAB^®^ software R2023b (The MathWorks, Inc., Natick, MA, United States). Specifically, we applied a second-order 6 Hz cut-off Butterworth filter, executing this filtration bi-directionally on the temporal data series to enhance data fidelity.

#### 2.4.2 Pain severity

To evaluate the severity of pain experienced by participants, the Numeric Rating Scale (NRS) was employed. This scale, ranging from 0 to 10, is utilized to quantify pain intensity, where a rating of 0 denotes “no pain” and a rating of 10 represents “the worst pain imaginable” (Nugent et al., 2021). This method provides a standardized approach for the subjective measurement of pain, facilitating the comparison of pain levels over time and across different conditions.

#### 2.4.3 Pressure pain threshold

To meticulously assess the general pain sensitivity of the participants, the Pressure Pain Threshold (PPT) was ascertained at two anatomically specific locations on the right side of the body, adhering to methodologies delineated in prior research dedicated to fibromyalgia (Rubio-Zarapuz et al., 2023; Rubio-Zarapuz et al., 2024). The designated anatomical sites for this evaluation were the lateral epicondyle and the medial fat pad proximal to the knee joint line. For the purpose of these measurements, an algometer was applied perpendicularly to the skin surface at each specified location. Participants were instructed to verbally signal the juncture at which the applied pressure transitioned to pain, thereby facilitating the determination of the PPT. The algometer employed in this investigation was fine-tuned to register pressure increments in gradations of 0.01 kilogram-force (kgf), a calibration that was strategically chosen to optimize the precision of the measurement while concurrently ensuring the comfort of the participants (Petzke et al., 2001; Kinser, Sands, and Stone, 2009). Moreover, the algometer underwent a stringent calibration process prior to the commencement of participant evaluations to affirm the measurement’s accuracy, as supported by previous research (Buranruk, 2022). This calibration was imperative to uphold the integrity and uniformity of the data amassed throughout the duration of the study.

### 2.5 Statistical analysis

In the statistical analysis section, we utilized SPSS (Statistical Package for Social Sciences, version 25, IBM, Armonk, NY, United States) for all analyses. Descriptive statistics were expressed as mean ± SD, and the normality of data distribution was verified using the Kolmogorov–Smirnov test. To evaluate the interventions’ effects, t tests were applied to parametric variables, and the Wilcoxon test was used for non-parametric variables. For comparing effects between groups, a one-way ANOVA was conducted. The significance level was maintained at *p* ≤ 0.05 for all tests. Furthermore, to provide a comprehensive understanding of the intervention impacts, we calculated effect sizes using Cohen’s d formula. This metric helps quantify the difference between two means in terms of standard deviation, facilitating an interpretation of practical significance. According to Cohen’s benchmarks, effect sizes are categorized as small (*d* = 0.2), medium (*d* = 0.5), and large (*d* = 0.8). These thresholds were applied to interpret the magnitude of the treatment effects observed in our study, ensuring a clear understanding of their clinical relevance.

### 2.6 Ethical aspects

The current study followed the ethical standards recognized by the Declaration of Helsinki, the EEC Good Clinical Practice recommendations (document 111/3976/88, July 1990), and current Spanish legislation regulating clinical and biomedical research in humans, personal data protection, and bioethics (Royal Decree 561/1993 on clinical trials and 14/2007, 3rd July, for Biomedical research). This research received formal approval from the University of Évora’s Research Ethics Committee, under the approval number 22033, dated 31 January 2022. Prior to commencement, the objectives and procedures of the study were thoroughly explained to all participants. Subsequently, participants voluntarily provided their agreement by signing an informed consent form, acknowledging their understanding and willingness to participate.

## 3 Results

Table 2 elucidates the descriptive statistical analysis of all variables quantified before and after the 1st, 8th, and 16th sessions across both intervention modalities. Notably, the NRS values exhibited a decrement of 1.2, 1.95, and 1.4 points at the 1st, 8th, and 16th session, respectively, for the Suit intervention. Conversely, the Exercise intervention revealed a reduction of 1.6, 0.8, and 2.44 points for each corresponding session. Furthermore, the PPT values indicated an enhanced tolerance to pain across all sessions within the Suit treatment, with the exception of the knee measurements during the second session (epicondyle: 0.37, 0.14, 0.86; knee: 0.46, 0.03, 0.46), and within the Exercise treatment (epicondyle: 0.25, 0, 0.28; knee: 0.38, 0.22, 0.99), barring the epicondyle measurements in the second session.

**TABLE 2:** Descriptive statistics.

Variables	Suit						Exercise					
	1st session		8th session		16th session		1st session		8th session		16th session	
	pre	post	pre	post	pre	post	pre	post	pre	post	pre	post
NRS, 0–10	6.00 ± 1.63	4.80 ± 1.69	6.80 ± 1.99	4.85 ± 1.49	5.30 ± 2.87	3.90 ± 2.42	6.90 ± 1.29	5.30 ± 1.77	6.50 ± 1.65	5.70 ± 1.83	6.33 ± 2.12	3.89 ± 2.76
PPT epicondyle, kg	1.80 ± 0.65	2.17 ± 0.49	1.86 ± 0.63	2.00 ± 1.02	1.81 ± 0.94	2.67 ± 1.98	2.09 ± 0.83	2.34 ± 1.25	2.21 ± 0.94	2.21 ± 0.86	2.19 ± 1.03	2.47 ± 0.96
PPT knee, kg	2.01 ± 0.69	2.47 ± 0.91	2.23 ± 1.55	2.26 ± 0.81	2.38 ± 1.44	2.84 ± 1.38	2.41 ± 1.31	2.79 ± 1.26	2.06 ± 0.74	2.28 ± 1.16	2.14 ± 1.23	3.13 ± 1.49
SmO_2_, %	59.44 ± 14.97	62.04 ± 16.31	55.49 ± 19.76	57.01 ± 15.59	55.27 ± 11.64	59.56 ± 15.36	49.59 ± 12.74	62.22 ± 12.16	48.05 ± 16.17	63.72 ± 14.49	65.48 ± 15.06	69.16 ± 14.05
THb, g/dL	11.77 ± 0.36	11.84 ± 0.40	11.81 ± 0.38	11.85 ± 0.33	11.89 ± 0.44	11.78 ± 0.48	11.84 ± 0.37	11.69 ± 0.34	11.76 ± 0.41	11.64 ± 0.39	11.74 ± 0.41	11.69 ± 0.43
O_2_Hb, g/dL	6.96 ± 1.66	7.3 ± 1.80	6.51 ± 2.20	6.73 ± 1.74	6.55 ± 1.29	6.97 ± 1.67	5.86 ± 1.48	7.25 ± 1.34	5.65 ± 1.89	7.42 ± 1.68	7.65 ± 1.63	8.05 ± 1.48
HHb, g/dL	4.8 ± 1.85	4.54 ± 2.08	5.3 ± 2.41	5.12 ± 1.90	5.35 ± 1.50	4.81 ± 1.94	5.98 ± 1.54	4.43 ± 1.48	6.11 ± 1.87	4.22 ± 1.67	4.09 ± 1.86	3.64 ± 1.72

Data are expressed as means ± SD for quantitative variables. NRS, Numeric Rating Scale; PPT, Pressure Pain Threshold; SmO_2_, Muscle Oxygen Saturation; THb, Total Hemoglobin; HHb, Deoxygenated Hemoglobin; O_2_Hb, Oxygenated Hemoglobin.

**FIGURE 1:**
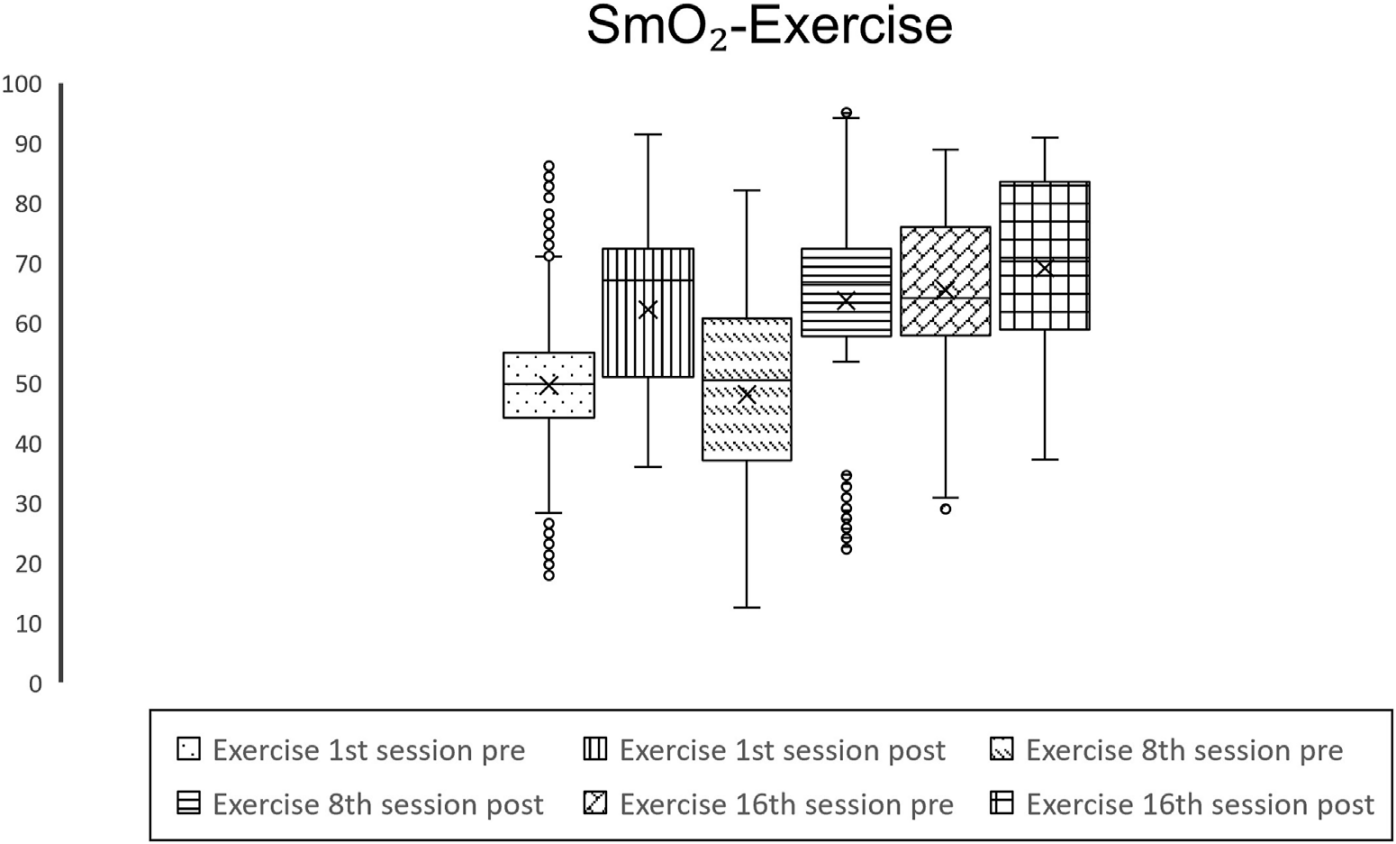
Graphical representation of values of SmO_2_ throughout the Suit intervention.

**FIGURE 2:**
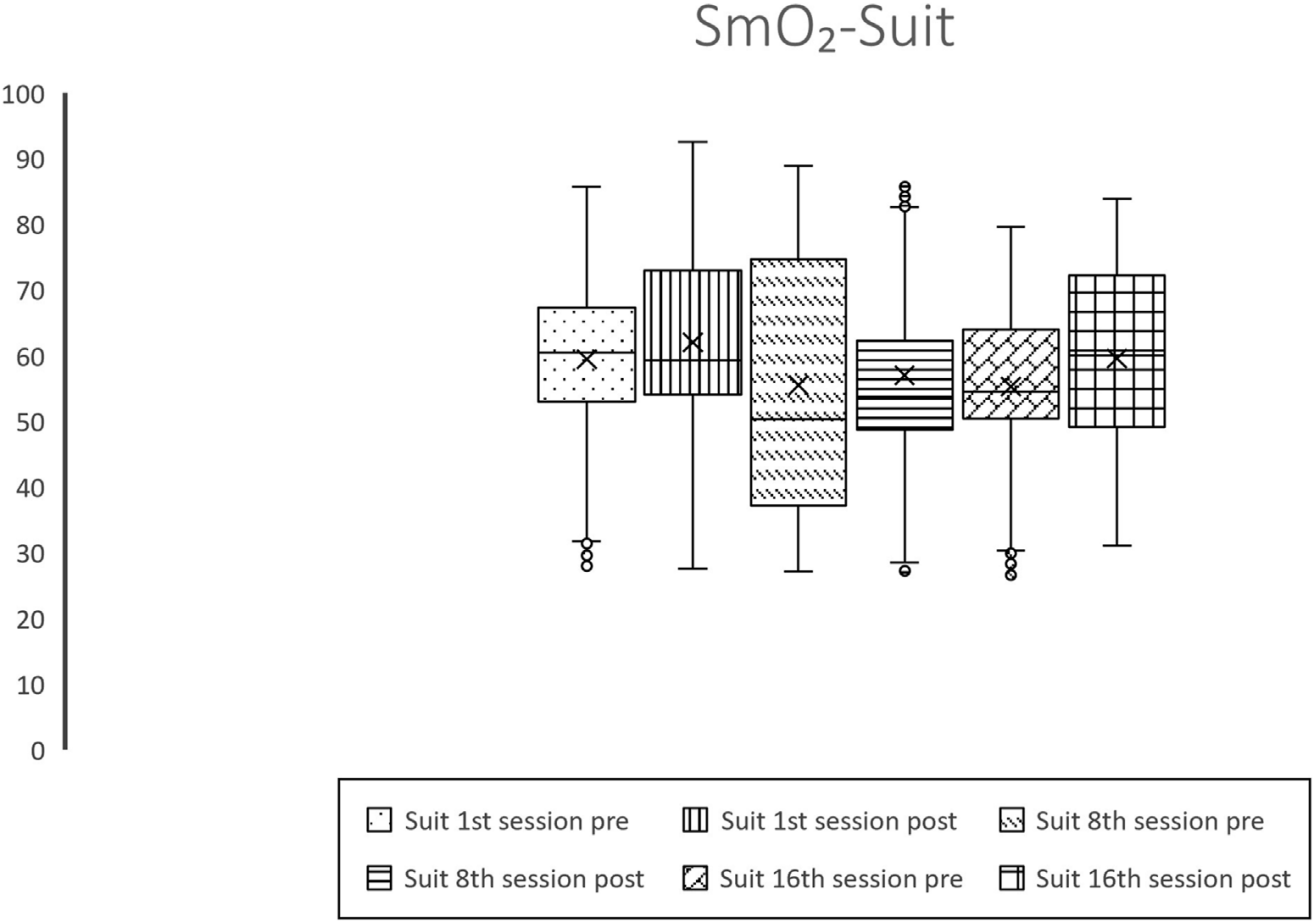
Graphical representation of values of SmO_2_ throughout the Exercise intervention.

In terms of muscle oxygenation metrics, no significant alterations were observed in THb levels; however, substantial elevations in SmO_2_ were recorded across all sessions for both treatments, with the Suit treatment noting increases of 2.6%, 1.52%, and 4.29%, and the Exercise treatment documenting enhancements of 12.63%, 15.67%, and 3.68%, as depicted in Figures 1, 2 and Table 2. Additionally, variations in oxygenated hemoglobin O_2_Hb and HHb were evident across all sessions for both intervention groups, with the Suit intervention registering an O_2_Hb augmentation of 0.34, 0.22, 0.42 g/dL and a HHb reduction of 0.26, 0.18, 0.54 g/dL, while the Exercise intervention showed an O_2_Hb increase of 1.39, 1.77, 0.4 g/dL and a HHb decrement of 1.55, 1.89, 0.45 g/dL. Moreover, baseline variations within the Exercise treatment demonstrated a 15.89% rise in SmO_2_, a 1.79 g/dL increase in O_2_Hb, and a 1.89 g/dL decrease in HHb between the 1st and 16th session which is worth mention.

Tables 3–5 present comparative statistics of the sample. On Table 3 we can see one-way ANOVA values presenting significant differences between all basal and post session muscle oxygenation variables measurements in both treatments. While also presenting significant differences between treatments in all measurements of muscle oxygenation variables except for the SmO_2_ and O_2_Hb values after the 1st session.

**TABLE 3:** Comparative statistics between groups.

Variables	Suit		Exercise		Pre 1st session		Post 1st session		Pre 8th session		Post 8th session		Pre 16th session		Post 16th session	
	Pre (*p*)	Post (*p*)	Pre (*p*)	Post (*p*)	*p*	ε^2^	*p*	ε^2^	*p*	ε^2^	*p*	ε^2^	*p*	ε^2^	*p*	ε^2^
NRS, 0–10	0.358	0.657	0.798	0.272	0.211	0.082	0.54	0.020	0.817	0.003	0.356	0.045	0.407	0.038	0.934	0.000
PPT epicondyle, kg	0.581	0.266	0.814	0.971	0.317	0.056	0.36	0.044	0.362	0.044	0.255	0.068	0.327	0.053	0.566	0.018
PPT knee, kg	0.814	0.614	0.549	0.097	0.743	0.006	0.65	0.011	0.705	0.008	0.732	0.006	0.743	0.006	0.806	0.003
SmO_2_, %	<.001*	<.001*	<.001*	<.001*	<.001*	0.146	0.052	0.001	<.001*	0.023	<.001*	0.090	<.001*	0.140	<.001*	0.098
THb, g/dL	<.001*	<.001*	<.001*	<.001*	<.001*	0.006	<.001*	0.045	<.001*	0.018	<.001*	0.131	<.001*	0.037	<.001*	0.007
HHb, g/dL	<.001*	<.001*	<.001*	<.001*	<.001*	0.149	<.001*	0.003	<.001*	0.027	<.001*	0.077	<.001*	0.150	<.001*	0.108
O_2_Hb, g/dL	<.001*	<.001*	<.001*	<.001*	<.001*	0.146	0.425	0.000	<.001*	0.017	<.001*	0.098	<.001*	0.133	<.001*	0.087

Table presents one-way ANOVA test results comparing all baseline and post-session measurements withing one treatment and comparing both treatments for each measurement. *p < 0.05. NRS, Numeric Rating Scale; PPT, Pressure Pain Threshold; SmO_2_, Muscle Oxygen Saturation; THb, Total Hemoglobin; HHb, Deoxygenated Hemoglobin; O_2_Hb, Oxygenated Hemoglobin.

Tables 3–5 elucidate the comparative statistical analyses of the sample under study. Specifically, Table 3 reveals the results of one-way ANOVA tests, indicating significant differences between all baseline and post-session measurements of muscle oxygenation variables within both treatment modalities. Additionally, significant disparities were observed between the treatments across all muscle oxygenation measurements, except for SmO_2_ and O_2_Hb values measured after the 1st session.

**TABLE 4:** Intrasession comparative statistics.

Variables	Suit						Exercise					
	1st session		8th session		16th session		1st session		8th session		16th session	
	*p*	Effect size	*p*	Effect size	*p*	Effect size	*p*	Effect size	*p*	Effect size	*p*	Effect size
NRS, 0–10	0.037*	−0.775	<.001*	−1.551	0.016*	−0.930	0.02*	−1.000	0.235	−0.402	0.002*	−1.467
PPT epicondyle, kg	0.03*	0.889	0.759	0.127	0.006*	1.000	0.284	0.422	1.000	−0.022	0.271	0.394
PPT knee, kg	0.049*	0.774	0.722	0.156	0.139	0.546	0.243	0.395	0.464	0.242	0.001*	1.623
SmO_2_, %	<.001*	0.229	<.001*	0.398	<.001*	0.441	<.001*	0.931	<.001*	0.884	<.001*	0.475
THb, g/dL	<.001*	0.338	0.752	0.009	<.001*	−0.382	<.001*	−0.675	<.001*	−0.427	<.001*	−0.297
O_2_Hb, g/dL	<.001*	0.263	<.001*	0.390	<.001*	0.390	<.001*	0.887	<.001*	0.894	<.001*	0.432
HHb, g/dL	<.001*	−0.201	<.001*	−0.421	<.001*	−0.462	0.027*	0.782	0.090	0.602	0.055	0.733

Table presents t test results comparing baseline and post-session measurement for each session and treatment. *p < 0.05. NRS, Numeric Rating Scale; PPT, Pressure Pain Threshold; SmO_2_, Muscle Oxygen Saturation; THb, Total Hemoglobin; HHb, Deoxygenated Hemoglobin; O_2_Hb, Oxygenated Hemoglobin.

**TABLE 5:** Intersession comparative statistics.

Variables	1st vs. 8th pre session		8th vs. 16th pre session		1st vs. 16th pre session		1st vs. 8th post session		8th vs. 16th post session		1st vs. 16th post session		1st pre vs. 8th post session		1st pre vs. 16th post session		8th pre vs. 16th post session	
	*p*	Effect size	*p*	Effect size	*p*	Effect size	*p*	Effect size	*p*		*p*	Effect size	*p*	Effect size	*p*	Effect size	*p*	Effect size
Suit Intervention																		
NRS, 0–10	0.411	−0.272	0.173	0.468	0.442	0.254	0.953	−0.019	0.343	0.316	0.31	0.34	0.186	0.453	0.035*	0.782	0.013*	0.979
PPT epicondyle, kg	0.441	−0.333	0.838	−0.0909	0.722	−0.156	0.232	0.455	0.057	−0.778	0.905	−0.0667	0.285	−0.422	0.015*	−0.933	0.241	−0.436
PPT knee, kg	0.529	−0.278	0.846	−0.0909	0.726	−0.167	0.441	0.255	0.119	−0.545	0.443	−0.254	0.296	−0.373	0.118	−0.584	0.333	−0.364
SmO_2_, %	<.001*	0.13	0.319	−0.0273	<.001*	0.46	<.001*	0.431	<.001*	−0.0958	<.001*	0.201	<.001*	0.538	<.001*	0.192	<.001*	−0.272
THb, g/dL	<.001*	−0.342	<.001*	−0.359	<.001*	−0.392	0.015*	.0.0752	0.021*	0.0663	0.277	0.0314	<.001*	−0.224	<.001*	−0.196	<.001*	0.181
O_2_Hb, g/dL	<.001*	0.121	0.012*	−0.0684	<.001*	0.452	<.001*	0.44	<.001*	−0.13	<.001*	0.202	<.001*	0.514	<.001*	0.171	<.001*	−0.294
HHb, g/dL	<.001*	−0.146	0.664	0.0119	<.001*	−0.465	<.001*	−0.39	0.033*	0.0613	<.001*	−0.189	<.001*	−0.488	<.001*	−0.189	<.001*	0.273
Exercise Intervention																		
NRS, 0–10	0.386	0.393	0.777	0.0975	0.63	0.2	0.565	−0.189	0.028*	0.891	0.243	0.421	0.085	0.618	0.037*	0.8	0.039*	0.823
PPT epicondyle, kg	0.307	−0.382	0.944	−0.0556	1	0	0.635	0.2	0.363	−0.389	1	0.0222	0.475	−0.273	0.294	−0.444	0.359	−0.378
PPT knee, kg	0.36	0.305	0.837	−0.0711	0.085	0.655	0.071	0.648	0.024*	−0.93	0.261	−0.403	0.619	0.163	0.009*	−1.14	0.033*	−0.859
SmO_2_, %	0.727	0.0096	<.001*	−0.981	<.001*	−0.891	0.033*	−0.0586	<.001*	−0.575	<.001*	−0.66	<.001*	−0.9	<.001*	−0.965	<.001*	−0.987
THb, g/dL	<.001*	0.482	<.001*	0.381	<.001*	0.615	<.001*	0.335	0.595	−0.0154	0.12	0.0452	<.001*	0.772	<.001*	0.877	<.001*	0.495
O_2_Hb, g/dL	0.496	0.0186	<.001*	−0.978	<.001*	−0.881	0.189	−0.0359	<.001*	−0.567	<.001*	−0.681	<.001*	−0.859	<.001*	−0.94	<.001*	−0.986
HHb, g/dL	0.635	0.013	<.001*	0.983	<.001*	0.889	0.006*	0.0745	<.001*	0.533	<.001*	0.643	<.001*	0.925	<.001*	0.975	<.001*	0.987

Table presents t test results comparing baseline and post-session measurements between different sessions within a same treatment. *p < 0.05. NRS, Numeric Rating Scale; PPT, Pressure Pain Threshold; SmO_2_, Muscle Oxygen Saturation; THb, Total Hemoglobin; HHb, Deoxygenated Hemoglobin; O_2_Hb, Oxygenated Hemoglobin.

Table 4 delineates comparative statistics between baseline and post-session measurements for each treatment, revealing significant variations in NRS values across all sessions for the Suit treatment, and for the 1st and 16th sessions within the Exercise treatment. Furthermore, PPT values at the epicondyle demonstrated significant differences for the Suit intervention in the 1st and 16th sessions, and PPT values at the knee for the 1st session within the Suit treatment and the 16th session within the Exercise treatment. Additionally, all muscle oxygenation variables exhibited significant differences between baseline and post-session evaluations, except for THb values in the 8^th^ session of the Suit treatment and HHb values for the 8th and 16th sessions in the Exercise treatment.

Table 5 presents comparative statistics between measurements, with NRS values showing significant differences between the baseline of the 1st session and the post-session measurement of the 16th session, as well as between the baseline of the 8^th^ session and the post-session measurement of the 16th session for both treatments. Significant variations were also observed in NRS values between the 8th and 16th post-session measurements for the Exercise treatment. Furthermore, PPT evaluations revealed significant disparities for epicondyle measurements between the baseline of the 1st session and the post-session measurement of the 16th for the Suit group. In contrast, PPT knee measurements exhibited significant differences between the 8th and 16th post-session measurements, the baseline of the 1st session and the post-session measurement of the 16th, and the baseline of the 8^th^ session and the post-session measurement of the 16th for the Exercise treatment. SmO_2_ values were significantly different in all comparisons between measurements, excluding the comparison between the 8th and 16th baseline measurements for the Suit treatment and between the 1st and 8^th^ baseline measurements for the Exercise treatment. Regarding THb, significant differences were noted in all comparisons between measurements, except between the 1st and 16th post-session measurements for the Suit treatment and between the 8th and 16th post-session measurements and the 1st and 16th post-session measurements for the Exercise treatment. Concerning O_2_Hb and HHb, all comparisons between measurements were significantly different, except for O_2_Hb between the 1st and 8^th^ sessions’ baseline as well as post-session measurements for the Exercise group, and for HHb between the 8th and 16th sessions’ baseline measurements for the Suit treatment, and the 1st and 8^th^ sessions’ baseline measurements in the Exercise treatment.

## 4 Discussion

The objective of this investigation was to elucidate the impacts of distinct therapeutic approaches on muscle oxygenation and pain modulation in patients diagnosed with Fibromyalgia. Post-research analysis supports the initial hypothesis, revealing that significant distinctions in muscle oxygenation and pain modulation parameters were observed for both treatment modalities under investigation.

In terms of pain modulation, there was a notable decrease in NRS values post-treatment across all sessions, apart from the 8th session in the Exercise treatment. These findings are in alignment with previous studies indicating a reduction in NRS scores subsequent to a 60-min neuromodulation session utilizing the EXOPULSE Mollii suit (Riachi et al., 2023; Rubio-Zarapuz et al., 2023; Rubio-Zarapuz et al., 2024). Moreover, the decrement in NRS values during the exercise regimen also corroborates earlier research (Rubio-Zarapuz et al., 2024), extending these outcomes to include HIIT sessions. This observation affirms that Fibromyalgia patients can not only complete HIIT sessions but also benefit from them when appropriately prescribed. Remarkably, a 2.44-point reduction in NRS scores was noted after the most rigorous HIIT session (16th session), surpassing the reductions reported in prior literature following strength training sessions in Fibromyalgia (Rubio-Zarapuz et al., 2024) and the initial exercise program session focused primarily on strength, which evidenced a 1.6-point decline.

Further on Analyses of NRS value progression throughout the treatment highlighted significant effect sizes (1st: 0.78, 8^th^: 1.55, 16th: 0.93) in the Suit treatment across sessions with the most substantial being observed in the 8th session. It is critical to note that the baseline NRS value was higher at the commencement of the 8th session, which may elucidate the observed larger effect size, even though the post-session value equaled that achieved in the initial session. The final session’s baseline NRS value was 5.3, reducing to 3.9 post-treatment, marking the lowest recorded value across the treatment span.

Moreover, where effect sizes were discerned to be 1 and 1.47 for the initial and terminal sessions, respectively. These sessions were distinguished by statistically significant differences, with the mean NRS value post-treatment in the final session approximating 3.89, closely mirroring the outcome observed in the final session of the Suit treatment. This pattern indicates an escalating efficacy in pain modulation as the intervention progresses, as evidenced by statistically significant disparities observed between the baseline measurements at the 1st and 8th sessions in comparison to the post-session measurement at the 16th session, with effect sizes of 0.78 and 0.98, respectively, for the Suit treatment, and 0.8 and 0.82 for the Exercise treatment. Additionally, within the Exercise treatment, significant differences were noted between the post-session measurements of the 8th and 16th sessions, revealing an effect size of 0.89. This delineates a consistent enhancement in the treatment’s impact on pain modulation over time.

In addition, it merits emphasis that no significant disparities were observed in the effects on pain modulation between the treatment modalities, as assessed by NRS values or PPT values across all evaluated sites. Nevertheless, a noteworthy finding emerged regarding PPT values measured at the epicondyle during the 1st and 16th sessions, exhibiting substantial effect sizes of 0.88 and 1, respectively, and at the knee during the initial session with an effect size of 0.77 in the Suit treatment. The escalation in effect size for epicondyle measurements suggests a trend analogous to that discerned in NRS values, contrasting with prior studies employing the same therapeutic approaches that did not report significant variations in PPT values at the epicondyle and knee (Rubio-Zarapuz et al., 2024). Furthermore, the PPT values at the knee, pre- and post-sixteenth session, revealed an effect size of 1.6, underscoring the premise that increased exercise intensities facilitate enhanced pain modulation outcomes. The absence of other significant differences in PPT measurements within the exercise treatment presents a stark contrast to significant outcomes in both the knee and epicondyle previously documented with strength training sessions (Rubio-Zarapuz et al., 2024), further substantiating the differential impact of varied exercise modalities on pain modulation.

Recent investigations have increasingly supported the hypothesis that mitochondrial dysfunction plays a pivotal role in Fibromyalgia (Shang et al., 2012; Gerdle et al., 2020; Villafaina et al., 2023), with emerging studies highlighting diminished levels of SmO_2_ among this patient demographic (Rubio-Zarapuz et al., 2023; Rubio-Zarapuz et al., 2024; Villafaina et al., 2023). This reduction in SmO_2_, as evidenced by our research, demonstrates that patients subjected to the Suit and Exercise treatments exhibited SmO_2_ percentages of 59.44% and 49.59%, respectively, a significant deviation from the normative value of 75% (Chen et al., 2020). Such diminished SmO_2_ levels could elucidate the compromised exercise tolerance observed in Fibromyalgia patients, given the critical role of oxygen in aerobic metabolism and ATP synthesis (Nakazawa et al., 2016). This inability to fully harness aerobic metabolism necessitates reliance on anaerobic processes, leading to acidosis, further exacerbated by prevalent sedentarism within this population, perpetuating a cycle of pain, inactivity, and fatigue. Our findings present a compelling narrative, showcasing statistically significant enhancements in SmO_2_ and O_2_Hb across all sessions for both therapeutic modalities. Additionally, a notable increase in total THb was observed, with the exception of the 8th session in the Suit treatment, alongside a reduction in HHb apart from the 8th and 16th sessions of the Exercise treatment. These results underscore the beneficial impacts of both treatments on muscle oxygenation, aligning with prior evidence (Rubio-Zarapuz et al., 2024; Rubio-Zarapuz et al., 2023). Distinctly, Table 3 illustrates statistically significant differences across all parameters between the treatments, excluding the O_2_Hb values post the initial session. These findings favor the Exercise treatment over the EXOPULSE Mollii Suit intervention in enhancing muscle oxygenation, as evidenced by the mean increases in SmO_2_ post-treatment in the exercise treatment (1st: 12.63%, 8th: 15.67%, 16th: 3.68%) in comparison to the Suit group (1st: 2.6%, 8th: 1.52%, 16th: 3.68%), mean O_2_Hb increment in the Exercise group (1st: 1.39, 8th: 1.77, 16th: 0.4) *versus* the Suit group (1st: 0.34, 8th: 0.22, 16th: 0.42), and HHb decrease in the Exercise group (1st: 1.55, 8th: 1.89, 16th: 0.45) compared to the Suit group (1st: 0.26, 8th: 0.18, 16th: 0.54). Notably, the observed increases in SmO_2_ within the Suit treatment did not surpass 5% for any session, albeit being statistically significant, the effect sizes remained modest (1st: 0.23, 8^th^: 0.4, 16th: 0.44), not reaching the medium effect size benchmark of 0.5. Conversely, the Exercise treatment demonstrated remarkable SmO_2_ enhancements during the initial (12.63%) and eighth sessions (15.67%), with effect sizes indicating large impacts (1st: 0.93, 8^th^: 0.88). However, the observed increase of 3.68% in SmO_2_ following the 16th session may initially imply a reduction in the effectiveness of the treatment over time. Nevertheless, a detailed examination reveals a different narrative. The baseline SmO_2_ mean value prior to the 16th session stood at 65.48%, marking a significant enhancement of 15.89% and 17.43% relative to the initial and eighth sessions’ baseline values, respectively, and yielding substantial effect sizes of 0.89 and 0.98. These observations unequivocally indicate that a structured training regimen, entailing two sessions per week of strength-based HIIT, elicits significant physiological adaptations within an 8-week period in female Fibromyalgia patients. These adaptations are characterized by elevated basal muscle oxygenation, as evidenced by increased SmO_2_ and O_2_Hb values alongside decreased HHb levels. Reaching this pivotal juncture, patients exhibit SmO_2_ levels merely 10% below the normative benchmark, a considerable improvement from the over 25% disparity noted prior to the commencement of the treatment. Notably, subsequent to the 16th session, patients achieved a mean SmO_2_ value of 69.14%, which closely approaches the standard reference values. This progression underscores the profound impact of the exercise regimen in fostering muscle oxygenation enhancements, suggesting its efficacy in addressing the oxygenation deficits inherent in Fibromyalgia patients.

Upon meticulous examination of the dataset, we can conclude that both the neuromodulation and exercise interventions exhibit considerable potential in the management of Fibromyalgia. Notably, each treatment demonstrated significant efficacy in modulating pain, as evidenced by the reduction in NRS scores to values below 4 upon the completion of the treatment regimen. Additionally, each session of both treatments resulted in significant improvements in muscle oxygenation parameters. Crucially, while enhancements in muscle oxygenation were observed following each treatment session, it was the Exercise treatment uniquely that induced basal adaptations in muscle oxygenation. This distinction underscores the Exercise treatment’s capacity to not only elicit immediate responses in muscle oxygenation but also to foster long-term physiological adaptations, enhancing the oxygenation baseline and potentially offering sustained benefits for individuals afflicted with Fibromyalgia.

### 4.1 Practical applications

This investigation introduces a paradigm shift for healthcare practitioners by elucidating two viable therapeutic modalities for Fibromyalgia management. The evidence indicates a discernible superiority of an 8-week Training regimen, entailing bi-weekly sessions of strength-based HIIT, in achieving sustained enhancements in both muscle oxygenation and pain modulation. Conversely, neuromodulation therapy utilizing the EXOPULSE Mollii Suit has also demonstrated effectiveness in augmenting muscle oxygenation and mitigating pain, albeit to a lesser extent compared to the exercise regimen.

Consequently, the integration of insights from this research enables clinicians to formulate customized treatment strategies that embrace a multimodal approach. Such approaches are finely tuned to meet the distinct requirements and preferences of individual patients, heralding a progressive step towards optimizing patient care. By deploying targeted interventions that comprehensively address the complex dimensions of Fibromyalgia, these personalized treatment plans hold the promise of fostering im-proved health outcomes and elevating the quality of life for those afflicted with the condition.

### 4.2 Limits of the study

A primary limitation of this investigation was the constrained sample size, a challenge exacerbated by the inherently sedentary lifestyle characteristic of patients diagnosed with this condition, which rendered the recruitment of participants willing to commit to the regimen a complex endeavor. Additionally, the absence of biochemical evaluations and more comprehensive neuromodulation assessments constitutes another significant limitation. These omitted analyses could have provided deeper insights into the physiological and neurobiological impacts of the treatment modalities on patients, thereby enhancing the understanding of their efficacy in managing Fibromyalgia.

### 4.3 Future lines of research

Further research regarding muscle oxygenation is necessary as research on the topic is still scarce. Future research should look on providing normative values for muscle oxygenation in Fibromyalgia patients as well as further research in non-pharmacological such as exercise treatments with greater samples as well as combined with nutritional therapy. Further on, research on the time the effects of training last on the organism of Fibromyalgia patients if the treatment is interrupted could be potentially beneficial. Moreover, research on the combined effects of both exercise and neuromodulation treatment are worth studying as both of them have proven beneficial in isolation.

Continued exploration into muscle oxygenation within the context of Fibromyalgia is imperative, given the current scarcity of research in this area. Future studies should endeavor to establish normative values for muscle oxygenation specific to Fibromyalgia patients. Additionally, there is a pronounced need for expanded investigations into non-pharmacological interventions, such as exercise treatments, utilizing larger participant cohorts and, potentially, integrating nutritional therapy to assess synergistic effects. Moreover, an examination of the durability of training effects on Fibromyalgia patients, particularly in scenarios where treatment is discontinued, would provide invaluable insights into the long-term viability of such interventions. Further on, investigating the collective impact of exercise and neuromodulation therapies, given their demonstrated efficacy as standalone treatments, also presents a promising avenue for future research. Such studies could illuminate potential synergies between these modalities, offering a more comprehensive approach to the management of Fibromyalgia.

## 5 Conclusion

In conclusion, this study demonstrates that both neuromodulation therapy, specifically utilizing the EXOPULSE Mollii Suit, and a structured exercise program are efficacious in modulating pain and enhancing muscle oxygenation in Fibromyalgia patients. Despite the absence of a significant difference in pain modulation efficacy between the two treatments, the exercise intervention exhibits a more profound impact on muscle oxygenation, facilitating long-term basal adaptations. This finding underscores the potential of exercise as a critical component in the holistic management of conditions involving altered muscle oxygenation and pain perception, such as Fibromyalgia.

Future studies should consider larger sample sizes to confirm these findings and explore the underlying mechanisms driving the differential effects of exercise and neuromodulation therapy on muscle oxygenation. Additionally, longitudinal studies examining the long-term benefits and potential synergistic effects of combining both treatment modalities could provide valuable insights into optimizing therapeutic strategies for Fibromyalgia management.

## Data Availability

The original contributions presented in the study are included in the article/Supplementary material, further inquiries can be directed to the corresponding author.
